# Optical Absorption on Electron Quantum-Confined States of Perovskite Quantum Dots

**DOI:** 10.3390/nano12172973

**Published:** 2022-08-28

**Authors:** Serhii I. Pokutnii, Andrzej Radosz

**Affiliations:** 1 Department of Theoretical Physics of Nanosystems, Chuiko Institute of Surface Chemistry of National Academy of Sciences of Ukraine, 17 General Naumov Str., 03164 Kyiv, Ukraine; 2 Department of Quantum Technologies, Wrocław University of Science and Technology, 27 Wybrzeże Wyspiańskiego, 50-370 Wrocław, Poland

**Keywords:** electron quantum-confined states, oscillator strengths, light absorption, quantum dots

## Abstract

In the framework of the dipole approximation, it is shown that in the perovskites quantum dots (QDs) ${\mathrm{FAPbBr}}_{3\;}\;$and {en} ${\mathrm{FAPbBr}}_{3\;}$ interacting with low-intensity light, the oscillator strengths of transitions, as well as the dipole moments allowing transitions between one-particle electron quantum-confined states, attain values considerably (by two orders of magnitude) exceeding the typical values of the corresponding quantities in semiconductors. It has been established that the maximum values of the cross-section optical absorption of perovskite QDs are reached at the resonant frequencies of electron transitions. This makes it possible to use such nanosystems as of strong absorption nanomaterials in a wide range of infrared waves.

## 1. Introduction

In recent years, interest has been growing in the study of electron states with a high binding energy and resistance to thermally driven dissociation in perovskite nanostructured materials. These electron states are intensively used in devices for controlling electron and exciton transport processes, photon storage, transitions at the heterointerface of an exciton transistor, photoconversion, and photoluminescence in perovskite nanostructured materials [1,2,3,4,5,6,7,8,9,10,11,12,13]. Over the past decade, in metal-halide perovskite solar cells, a significant increase in energy conversion efficiency from 3.9% to 25.5% was observed [5,6,7,8,9,10,11,12,13,14]. This is of particular interest, as perovskite hybrid-chemical solar cells have grown recently into the promising candidate for wide commercial applications in photovoltaics [14,15,16].

Photovoltaic devices based on perovskite single crystals are emerging as a viable alternative to polycrystalline materials. Perovskite single crystals indeed possess lower trap state densities, higher carrier mobilities, longer diffusion lengths, and, potentially, can achieve higher performance with respect to those fabricated with polycrystalline films, although their integration in a complete device needs particular attention, as does the use of specifically tailored growth techniques [14,15,16,17]. In [18], the dependence of the photoconductivity of colloidal PbS/MA${\mathrm{PbBr}}_{3\;}$quantum dots in nanosized gaps between gold electrodes on light intensity was experimentally discovered. In [19], new active nanomaterials for nanolaser devices with amplified spontaneous emission were studied. The threshold of enhanced spontaneous emission in thin nanofilms of mixtures of polymer dyes and lead halide perovskites was studied. The mode of excitation of the early stage of amplified spontaneous emission in such nanolasers was discovered.

In [20], using first-principles calculations, the geometric and electronic structure of an organic–inorganic hybrid perovskite${\;\mathrm{FAPbX}}_{3\;}$(FA = CH (NH2)2 +; X = Cl, Br, I) was studied. Since the organic molecule in the center of the 3D hybrid perovskite is the key for its characteristics, the band gap of${\;\mathrm{FAPbX}}_{3\;}$was compared with the band gap of ${\;\mathrm{MAPbX}}_{3\;}$(MA = CH3NH3+) in Ref. [18]. In this case, the band gap of the perovskite${\;\mathrm{FAPbX}}_{3\;}$ turned out to be smaller than that of the perovskite${\;\mathrm{MAPbX}}_{3\;}$. Particularly, the calculated band gap of 1.40 eV${\;\mathrm{FAPbX}}_{3\;}$obtained in [20] correlated well with the value of the band gap of 1.41 eV established under the experimental conditions.

Perovskites containing the colloidal QDs ${\mathrm{FAPbBr}}_{3\;}$and {en}${\mathrm{FAPbBr}}_{3\;}$are promising newcomer optoelectronic materials. Colloidal QDs $\left\{\mathrm{en}\right\}{\mathrm{FAPbBr}}_{3\;}$ contain perovskite {en}${\mathrm{FAPbBr}}_{3.\;}$ The {en}${\mathrm{FAPbBr}}_{3\;}$perovskite was obtained by bulk doping ${\mathrm{FAPbBr}}_{3\;}$ perovskite with ethylenediammonium {en}. These nanomaterials have been used as highly absorbent nanolayers in solar cells. At the same time, such new optoelectronic materials have attracted increased attention because of their high energy conversion efficiency, reaching about 20% [15]. Nanosystems, composed of the colloidal QDs ${\mathrm{FAPbBr}}_{3\;}$and QDs {en}${\mathrm{FAPbBr}}_{3\;}$, belong to a new and developing class of nanomaterials in which, with the help of cation engineering, the bandgaps vary depending on the composition and size of the QDs of the lead-halide [13,21,22,23,24,25].

In [13], nanosystems consisting of the colloidal QDs ${\mathrm{FAPbBr}}_{3\;}$and QDs {en}${\mathrm{FAPbBr}}_{3\;}$ were experimentally studied. It was found that bulk doping${\mathrm{FAPbBr}}_{3\;}$perovskite with ethylenediammonium {en} led to an increase in the bandgap in the QDs {en}${\mathrm{FAPbBr}}_{3\;}$. This caused an increase in the photoluminescence-lifetimes in the QDs {en}${\mathrm{FAPbBr}}_{3\;}$ compared with the photoluminescence-lifetimes in the QDs ${\mathrm{FAPbBr}}_{3\;}$.

In [13], photoluminescence-lifetimes were also estimated in the QDs${\;\mathrm{FAPbBr}}_{3\;}$and QDs {en}${\;\mathrm{FAPbBr}}_{3\;}$. It was shown that the photoluminescence-lifetimes in the QDs ${\mathrm{FAPbBr}}_{3\;}$and QDs {en}${\;\mathrm{FAPbBr}}_{3\;}$were formed by allowed electron transitions between the quantum-confined energy levels arising in QDs perovskites.

Optical absorption of perovskite QDs${\;\mathrm{FAPbBr}}_{3\;}$and QDs {en}${\;\mathrm{FAPbBr}}_{3\;}$is poorly studied. In particular, the nature of strong absorption in perovskite QDs${\;\mathrm{FAPbBr}}_{3\;}$and QDs {en}${\;\mathrm{FAPbBr}}_{3\;}$is not clear. Therefore, in the present work, in the framework of the dipole approximation, the intraband optical absorption due to allowed transitions between one-particle electron quantum-confined states emerging in the QDs${\;\mathrm{FAPbBr}}_{3\;}$is investigated.

The addition of the mentioned above QDs can increase the efficiency of perovskite solar cells by up to 34% [15]. By the appropriate tailoring of nano-admixtures it is, however, possible to pass the Shockley–Queisser efficiency limit in these cells. Using metallic nanocomponents, even a 40% relative increase of the efficiency of a perovskite cell has been experimentally demonstrated [16]. This is even larger than in conventional (Si or CIGS) cells [21], and reflects the fact that in chemical cells without a p-n junction a different plasmonic photovoltaic effect dominates [26,27,28]. The simultaneous application of QDs would also result in the further increase of the efficiency and in completely yet-unexplored synergetic effect of QD metallic nanoparticle complexes. The coupling of excitons in QDs with surface plasmons in metallic nanoparticles sensitive to the proximity of both subsystems would beneficially influence onto both nano-agents in a different way than separately. This additionally motivates the presented study.

## 2. Theoretical Method and Model

### 2.1. Quantum-Confined Electron States in a Nanosystem

In the experimental work [13], there were studied QDs of perovskites ${\mathrm{FAPbBr}}_{3\;}$and $\left\{\mathrm{en}\right\}{\mathrm{FAPbBr}}_{3\;}.$ It was assumed that the QDs were spherical with average radii *a* = 5.5 nm. The values of broadenings of the absorption edge $\;∆E_{1}$ = 71 meV for the QD ${\mathrm{FAPbBr}}_{3\;}$ and$\;∆E_{2}$ = 120 meV for QD $\left\{\mathrm{en}\right\}{\mathrm{FAPbBr}}_{3\;}$were determined. In the QD perovskites ${\mathrm{FAPbBr}}_{3\;}$and in the QD perovskites {en}${\mathrm{FAPbBr}}_{3\;}$, the dielectric constant *ε*, the effective electron masses $m_{e\;}$, and the bandgap $\;E_{g},\;$respectively, were:$\;\varepsilon =8.6,\;m_{e\;}=$ 0.26 $m_{0},$ $\;E_{g}=$ 2.34 eV and $\varepsilon =7,\;m_{e\;}=$ 0.21 $m_{0},$ $\;E_{g}=$ 2. 43 eV ($m_{0}\;$is the mass of a free electron).

In [13], the QD was modeled by a spherical potential well with infinitely deep walls. In this case, the energy levels of an electron (n,l) were determined by the formula [29]:

(1) $$E_{n,l}\left(a\right)=\frac{\hbar^{2}}{\;2m_{e\;}a^{2}{}_{\;}}\;{\left(X_{n,l}\right)}^{2}$$

where the subscripts (*n, l*) are the principal and azimuthal quantum numbers for the electron and $X_{n,l}$ are the roots of the Bessel function, i.e.,$\;J_{l+1/2}$ ($X_{n,l})$ = 0. The energy levels of an electron $\;E_{n,l}\left(a\right)$ (1) were in the conduction band QD. The energy levels (1) of an electron were obtained in [29] under the assumption that the conduction band QD had a parabolic shape. This was carried out if the band nonparabolicity parameter *η*(*a*) for electronic energy levels (1) in a QD of radius *a* satisfied the condition

(2) $$\eta (a)=\left(E_{n,l}\left(a\right)-\;E_{g}\right)\;/\;E_{g}\leq 0.1$$

where $\;E_{g}$ is the QD bandgap.

Assuming that the quantum-confined levels of the electron energy $\;E_{n,l}\left(a\right)$ (1) of the QD are only slightly broadened at temperature *T*, the energy separation between the levels is

(3) $$∆E_{n,l}\left(a\right)=E_{n,l+1}\left(a\right)–E_{n,l}\left(a\right)\ll kT$$

When condition (3) is satisfied, the electron states (1) in QD can be observed. These electron states (1) can be described by the wave functions of an electron in a spherical quantum well with infinitely high walls [29].

**Figure 1:**
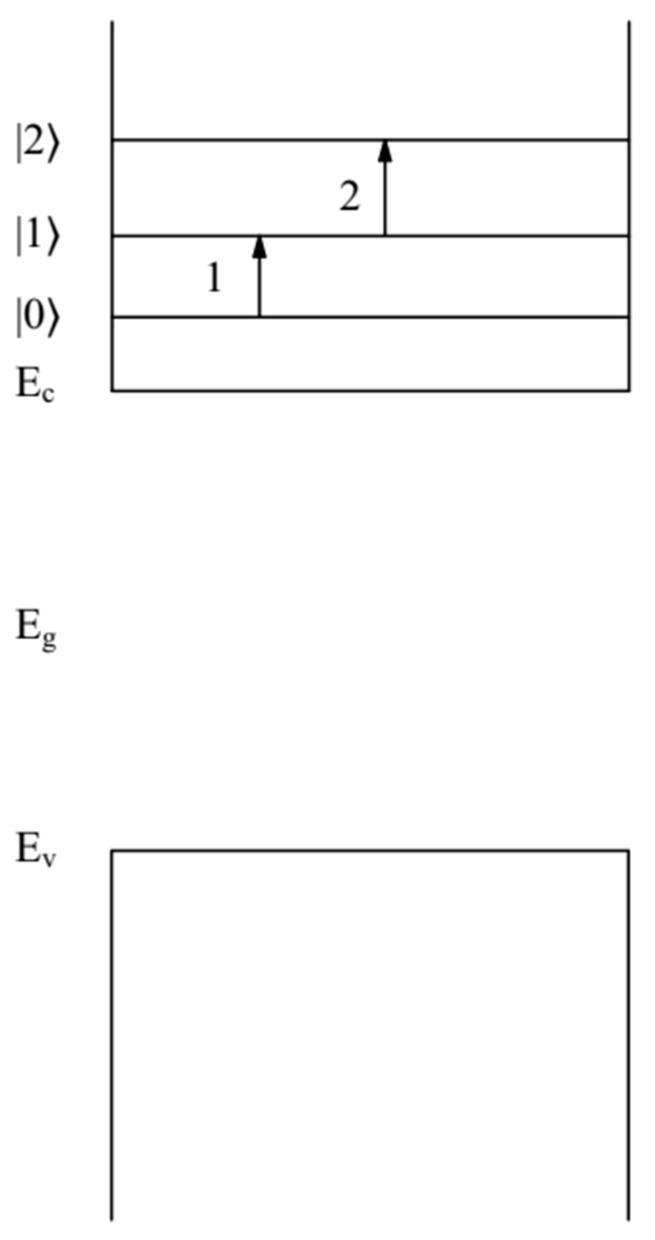
Band diagram of the QD ${\mathrm{FAPbBr}}_{3.\;}$ Quantum-confined energy levels |0〉$=E_{1,0,0}=48\;\mathrm{meV},$ |1〉$=E_{1,1,1}=99.2\;\mathrm{meV},\;$ |2〉$=E_{1,2,0}=164.6\;\mathrm{meV}$. Electrons are in the conduction band of QD ${\mathrm{FAPbBr}}_{3\;}.\;$ Arrows show electron transitions: electron transition (1) between levels |0〉 and |1〉 (the energy of such a transition is $∆E_{1,0,0}^{1,1,1}\left(a\right)$ = 51.2 meV), electron transition, and (2) between levels |1〉 and |2〉 (the energy of such a transition is $∆E_{1,1,1}^{1,2,0}\left(a\right)=65.4\;\mathrm{meV})$. The energies$\;E_{c}$, $\;E_{v}$ and $\;E_{g}$ = 2.34 eV correspond to the positions of the bottom of the conduction band and the top of the valence band, and the bandgap QD ${\mathrm{FAPbBr}}_{3\;}$, respectively.

In [13], it was shown that the broadening $\;∆E_{1}$ = 71 meV of the absorption edge in the QD ${\mathrm{FAPbBr}}_{3\;}$ was caused by two intraband allowable transitions between the quantum-confined states of an electron (n=1,l=0,t=0) and (n=1,l=1,t=1 ), as well as (n=1,l=1,t=1 ) and (n=1,l=2,t=0 ) (where *t* is the magnetic quantum number of the electron). Such transitions were allowed by the selection rules. The energies of these electron levels, according to Formula (1), were $\;E_{1,0,0}=48\;\mathrm{meV}$,$\;E_{1,1,1}=99.2\;\mathrm{meV}$, and$\;E_{1,2,0}=164.6\;\mathrm{meV}.\;$The transition energies between these electron levels were $∆E_{1,0,0}^{1,1,1}\left(a\right)$ = 51.2 meV and $∆E_{1,1,1}^{1,2,0}\left(a\right)$ = 65.4 meV (see Figure 1). The broadening value of $\;∆E_{1}$ = 71 meV of the absorption edge in the QDs ${\mathrm{FAPbBr}}_{3\;}$with an accuracy not exceeding 14% was caused by these transitions [13].

**Figure 2:**
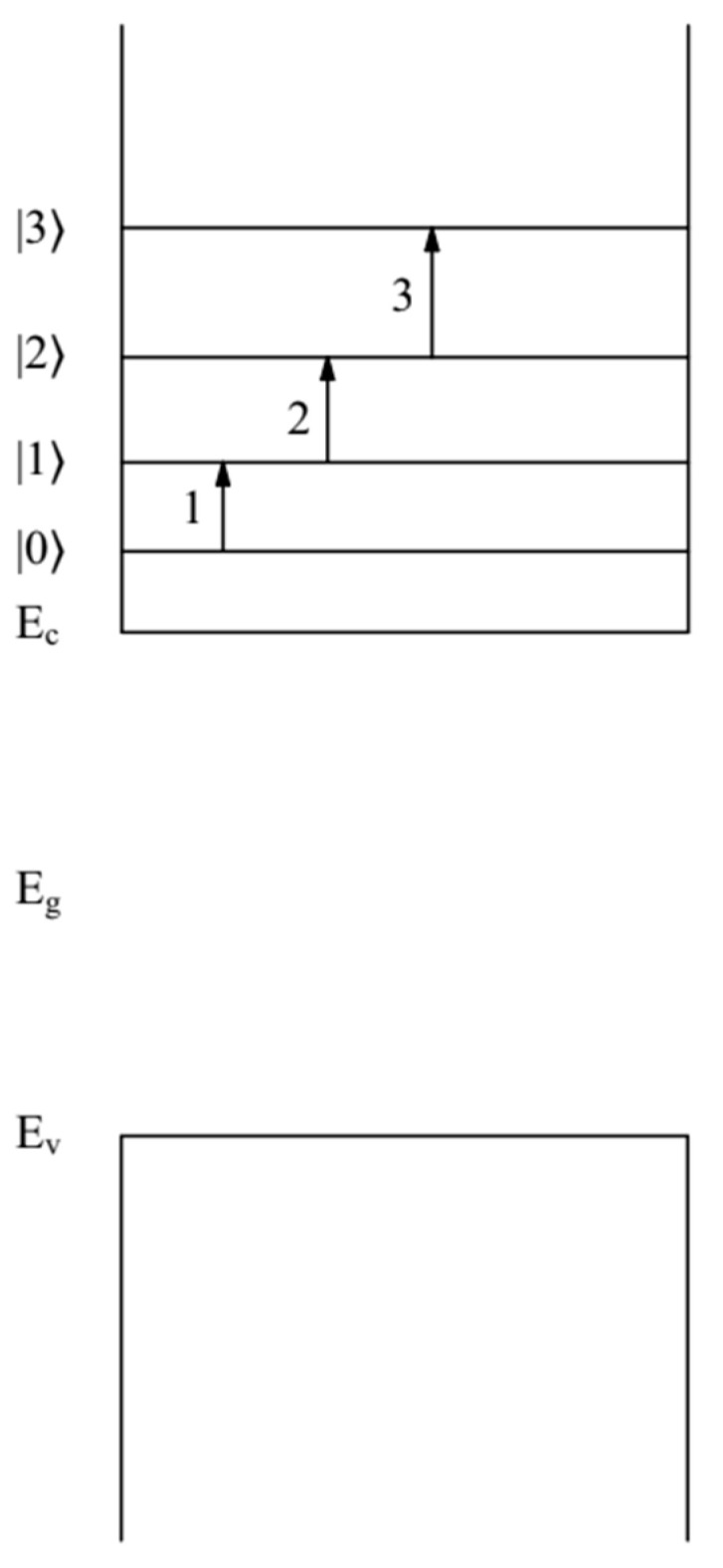
Band diagram of the QD $\{\mathrm{en}\}{\mathrm{FAPbBr}}_{3\;}.$ Quantum-confined energy levels |0〉$=E_{1,0,0}=58\;\mathrm{meV},$ |1〉$=E_{1,1,1}=122\;\mathrm{meV},\;$ |2〉$=E_{1,2,0}=204\;\mathrm{meV},$ |3〉$=E_{1,3,1}=316\;\mathrm{meV}.$ Electrons are in the conduction band of QD $\{\mathrm{en}\}{\mathrm{FAPbBr}}_{3\;}.$ Arrows show electron transitions: electron transition (1) between levels |0〉 and |1〉 (the energy of such a transition is $∆E_{1,0,0}^{1,1,1}\left(a\right)$ = 64 meV), electron transition (2) between levels |1〉 and |2〉 (the energy of such a transition is $∆E_{1,1,1}^{1,2,0}\left(a\right)=82\;\mathrm{meV})$, electron transition (3) between levels |2〉 and |3〉 (the energy of such a transition is $∆E_{1,2,0}^{1,3,1}\left(a\right)$ = 112 meV). The energies$\;E_{c}$, $\;E_{v},$ and $\;E_{g}$ = 2.43 eV correspond to the positions of the bottom of the conduction band, the top of the valence band, and the bandgap QD $\{\mathrm{en}\}{\mathrm{FAPbBr}}_{3\;}$, respectively.

Doping led to a change in the optical characteristics of the QD $\{\mathrm{en}\}{\mathrm{FAPbBr}}_{3\;}$ compared with the optical characteristics of the $\mathrm{QD}\;{\mathrm{FAPbBr}}_{3\;}$ [13]. In this case, the broadening of the$\;∆E_{2}$ = 120 meV absorption edge in the QD $\{\mathrm{en}\}{\mathrm{FAPbBr}}_{3\;}$was caused by three intraband allowable transitions between the quantum-confined states of an electron (n=1,l=0,t=0) and (n=1,l=1,t=1 ), (n=1,l=1,t=1 ) and (n=1,l=2,t=0 ), as well as (n=1,l=2,t=0 ) and (n=1,l=3,t=1 ). Such transitions were allowed by the selection rules. The energies of these electron levels, according to formula (1), were$\;E_{1,0,0}=58\;\mathrm{meV},$ $\;E_{1,1,1}=122\;\mathrm{meV}$,$\;E_{1,2,0}=204\;\mathrm{meV}$, and$\;E_{1,3,1}=316\;\mathrm{meV}.$ The transition energies between these electron levels were $∆E_{1,0,0}^{1,1,1}\left(a\right)$ = 64 meV, $∆E_{1,1,1}^{1,2,0}\left(a\right)\;$= 82 meV and $∆E_{1,2,0}^{1,3,1}\left(a\right)$ = 112 meV, correspondingly. The broadening value of $\;∆E_{2}=120\;\mathrm{meV}\;$of the absorption edge in the QDs $\left\{\mathrm{en}\right\}{\mathrm{FAPbBr}}_{3\;}$, with an accuracy not exceeding 10%, was caused by these transitions [13] (see Figure 2). These transition energies were obtained in [13] using formula (1). Condition (2) for electron states (1) in QDs was fulfilled.

### 2.2. Dipole Moments of Transitions in a Nanosystem

The quantum-confined states of electron (n, l) (1) in QDs in the field of a light wave was adequately described in the dipole approximation [30,31]. Let us write an expression for the dipole moments of the intraband allowable transitions $D_{n,l,t}^{n,l+1,t}$(*a*) between the quantum-confined states of an electron (n=1,l,t) and (n=1,l+1,t) in QDs (where the azimuthal quantum number of the electron is l≤2, and the magnetic quantum number of the electron is t=0,1):

(4) $$D_{1,l,t}^{1,l+1,t}(a)\;=\;\Psi_{1,l+1,t}\left(r,\theta \right)|D\left(r\right)|\Psi_{1,l,t}\left(r,\theta \right)$$

where the operator of the dipole moment of the electron located in the QD is expressed as [32]

(5) D(r) = Λer

In Formula (4), the nanosystem parameter

(6) $$\;\Lambda =3\varepsilon_{0}/(2\varepsilon_{0}+\varepsilon )$$

(here $\varepsilon_{0}\;\mathrm{is}\;\mathrm{the}\;\mathrm{permittivity}\;\mathrm{matrix}\;\mathrm{and}\;\varepsilon_{0}=1),\;$***r*** is the radius vector determining the distance between the electron and the center of the QD and *θ* is the azimuthal angle defining the position of electron radius vector. In Formula (3), the states |n=1,l,t〉 and |n=1,l+1,t〉 electron are described by the electron wave functions $\Psi_{1,l,t}\left(r,\theta \right)$ and $\Psi_{1,l+1,t}\left(r,\theta \right)$ of an infinitely deep spherical potential well. Let us write explicitly the electron wave functions $\Psi_{1,l,t}\left(r,\theta \right)$ and $\Psi_{1,l+1,t}\left(r,\theta \right)$ for the states (n=1,l,t) (where l≤2, t=0, 1) [33,34,35]. To simplify notation, let: α=1,1; β=1,2; σ=1,3, then:

(7) $$\Psi_{1,0,0}\left(r,\theta \right)=\frac{\pi^{-\frac{1}{2}}a^{-\frac{3}{2}}j_{0}\left(\pi \frac{r}{a}\right)}{j_{1}\left(\pi \right)}$$

(8) $$\Psi_{1,1,1}\left(r,\theta ,\varphi \right)=\frac{{\left(\frac{3}{2\pi }\right)}^{\frac{1}{2}}a^{-\frac{3}{2}}\exp\left(i\varphi \right)\sin\left(\theta \right)j_{1}\left({Χ}_{\alpha }\frac{r}{a}\right)}{j_{2}\left(X_{\alpha }\right)}$$

(9) $$\Psi_{1,2,0}\left(r,\theta ,\varphi \right)=\frac{{\left(\frac{5}{4\pi }\right)}^{\frac{1}{2}}a^{-\frac{3}{2}}\left(3\cos^{2}\left(\theta \right)-1\right)j_{2}\left({Χ}_{\beta }\frac{r}{a}\right)}{j_{3}\left(X_{\beta }\right)}$$

(10) $$\Psi_{1,3,1}\left(r,\theta ,\varphi \right)=\frac{{\left(\frac{7}{3\pi }\right)}^{\frac{1}{2}}a^{-\frac{3}{2}}\exp\left(i\varphi \right)\left(5\;\cos^{4}\left(\theta \right)-3\;\cos^{2}\left(\theta \right)\right)j_{3}\left({Χ}_{\sigma }\frac{r}{a}\right)}{2j_{4}\left(X_{\sigma }\right)}$$

where $\;j_{n\;}\left(X\right)$ are the spherical Bessel functions and φ are the polar angle defining the position of electron radius vector. We can integrate expression (4), taking into account (5) and (6)–(10). As a result, we get expressions that define the values for the dipole moments of the allowable transitions $D_{n,l,t}^{n,l+1,t}$(*a*) between the quantum-confined states of an electron (n=1,l,t) and (n=1,l+1,t) (where l≤2, t=0, 1) in QDs:

(11) $$D_{1,0,0}^{1,1,1}\left(a\right)=\frac{2^{\frac{3}{2}}\pi \Lambda ea}{\sqrt{3}X_{\alpha }j_{2}\left(X_{\alpha }\right)\left(X_{\alpha }^{2}-\pi^{2}\right)}*\left(\cos X_{\alpha }-\frac{\left(3X_{\alpha }^{2}-\pi^{2}\right)\sin X_{\alpha }}{X_{\alpha }\left(X_{\alpha }^{2}-\pi^{2}\right)}\right)$$

(12) $$D_{1,1,1}^{1,2,0}\left(a\right)=\frac{2^{3}\Lambda ea}{\sqrt{15}X_{\alpha }X_{\beta }j_{2}\left(X_{\alpha }\right)j_{3}\left(X_{\beta }\right)\left(X_{\beta }^{2}-X_{\;\alpha }^{2}\right)}*\frac{1}{X_{\alpha }X_{\beta }^{2}}*\left[\cos\left(X_{\alpha \beta }\right)-\cos\left(∆X_{\alpha \beta }\right)+\frac{\left(2+\pi \right)}{2}∆X_{\alpha \beta }-\frac{\left(\pi -2\right)}{2}X_{\alpha \beta }+2∆X_{\alpha \beta }\sin\left(∆X_{\alpha \beta }\right)+\frac{\sin\left(X_{\alpha \beta }\right)+\sin\left(∆X_{\alpha \beta }\right)}{2X_{\alpha }X_{\beta }}+\frac{\sin\left(X_{\alpha \beta }\right)-X_{\alpha \beta }\cos\left(∆X_{\alpha \beta }\right)}{2X_{\alpha \beta }^{2}}+\frac{\sin\left(X_{\alpha \beta }\right)-∆X_{\alpha \beta }\cos\left(∆X_{\alpha \beta }\right)}{2{\left(∆X_{\alpha \beta }\right)}^{2}}\right]$$

(13) $$D_{1,2,0}^{1,3,1}\left(a\right)=\frac{12\Lambda ea}{5\sqrt{7}X_{\beta }X_{\sigma }j_{3}\left(X_{\beta }\right)j_{4}\left(X_{\sigma }\right)\left(X_{\sigma }^{2}-X_{\beta }^{2}\right)}*\left(D_{1}\left(a\right)+D_{2}\left(a\right)+D_{3}\left(a\right)\right)$$

(14) $$D_{1}\left(a\right)=\mathrm{si}\left(∆X_{\beta \sigma }\right)+\frac{\sin\left(∆X_{\beta \sigma }\right)}{{\left(∆X_{\beta \sigma }\right)}^{2}}+\frac{\cos\left(∆X_{\beta \sigma }\right)}{∆X_{\beta \sigma }}-\frac{2\cos\left(∆X_{\beta \sigma }\right)}{{\left(∆X_{\beta \sigma }\right)}^{3}}+\frac{X_{\beta \sigma }^{3}}{X_{\beta }^{2}X_{\sigma }^{3}}\left(\frac{2\cos\left(∆X_{\beta \sigma }\right)}{X_{\beta \sigma }^{3}}-\frac{\sin\left(∆X_{\beta \sigma }\right)}{X_{\beta \sigma }^{2}}-\frac{\cos\left(X_{\beta \sigma }\right)}{X_{\beta \sigma }}-\mathrm{si}\left(X_{\beta \sigma }\right)\right)+\frac{X_{\beta \sigma }^{2}}{X_{\beta }^{2}X_{\sigma }^{2}}\left(\frac{\cos\left(X_{\beta \sigma }\right)}{X_{\beta \sigma }}+\frac{\sin\left(X_{\beta \sigma }\right)}{X_{\beta \sigma }^{2}}+\mathrm{si}\left(X_{\beta \sigma }\right)\right)$$

(15) $$D_{2}\left(a\right)=-\frac{3\Delta X_{\beta \sigma }^{2}}{X_{\beta }^{2}X_{\sigma }^{2}}\left(\frac{\cos\left(∆X_{\beta \sigma }\right)}{∆X_{\beta \sigma }}+\frac{\sin\left(∆X_{\beta \sigma }\right)}{∆X_{\beta \sigma }^{2}}+\mathrm{si}\left(∆X_{\beta \sigma }\right)\right)-\frac{3∆X_{\beta \sigma }}{X_{\beta }^{2}X_{\sigma }}\left(\frac{\cos\left(∆X_{\beta \sigma }\right)}{∆X_{\beta \sigma }}+\mathrm{si}\left(∆X_{\beta \sigma }\right)\right)+\left(\frac{\cos\left(∆X_{\beta \sigma }\right)}{∆X_{\beta \sigma }}+\mathrm{si}\left(∆X_{\beta \sigma }\right)\right)+\frac{3X_{\beta \sigma }}{X_{\beta }^{2}X_{\sigma }}\left(\frac{\cos\left(∆X_{\beta \sigma }\right)}{X_{\beta \sigma }}+\mathrm{si}\left(X_{\beta \sigma }\right)\right)+\mathrm{si}\left(X_{\beta \sigma }\right)-\mathrm{si}\left(∆X_{\beta \sigma }\right)+\frac{3\Delta X_{\beta \sigma }^{2}}{X_{\beta }X_{\sigma }^{3}}\left(\mathrm{si}\left(∆X_{\beta \sigma }\right)+\frac{\sin\left(∆X_{\beta \sigma }\right)}{∆X_{\beta \sigma }^{2}}+\frac{\cos\left(∆X_{\beta \sigma }\right)}{∆X_{\beta \sigma }}\right)$$

(16) $$D_{3}\left(a\right)=\frac{3\left(\sin\left(X_{\beta \sigma }\right)-\sin\left(∆X_{\beta \sigma }\right)\right)}{{Χ}_{\sigma }^{2}}+\frac{3\sin\left(∆X_{\alpha \beta }\right)}{{Χ}_{\sigma }∆X_{\beta \sigma }}-\frac{3\sin\left(X_{\alpha \beta }\right)}{{Χ}_{\sigma }X_{\beta \sigma }}+\frac{\left(X_{\beta \sigma }\cos\left(X_{\beta \sigma }\right)-\sin\left(X_{\beta \sigma }\right)\right)}{2X_{\beta \sigma }^{2}}+\frac{\left(\Delta X_{\beta \sigma }\cos\left(\Delta X_{\beta \sigma }\right)-\sin\left(\Delta X_{\beta \sigma }\right)\right)}{2\Delta X_{\beta \sigma }^{2}}$$

where $X_{\alpha \beta \;}=X_{\alpha \;}+X_{\beta \;}$; $X_{\beta \sigma \;}=X_{\beta \;}+X_{\sigma \;}\;$and $∆X_{\alpha \beta \;}$ $=X_{\beta \;}-X_{\alpha }$; $∆X_{\beta \sigma \;}$ $=X_{\sigma \;}-X_{\beta }$, *si*(*y*) is the integral sinus.

The oscillator strengths of the allowable intraband transitions $f_{n,l,t}^{n,l+1,t}$(*a*) between the quantum-confined states of an electron (n=1,l,t) and (n=1,l+1,t) can be represented as:

(17) $$f_{1,l,t}^{1,l+1,t}\left(a\right)=\left(X_{1,l+1}^{2}-X_{1,l}^{2}\right){\left|\frac{D_{1,l,t}^{1,l+1,t}\left(a\right)}{ea}\right|}^{2}$$

### 2.3. Intensity of Optical Transitions in the Nanosystem

Intensity$\;I_{1,l,t}^{1,l+1,t}\left(a\right),$ caused by the dipole-allowed intraband electron transitions between the quantum-confined states (n=1,l,t) and (n=1,l+1,t), is determined by the square of the overlap integral of the electron wave functions $\Psi_{1,l,t}\left(r,\theta \right)$ and $\Psi_{1,l+1,t}\left(r,\theta \right)$. [29,30]:

(18) $$I_{1,l,t}^{1,l+1,t}\left(a\right)=C{\left|\overset{2\pi }{\underset{0}{\int }}d\varphi \;\overset{\pi }{\underset{0}{\int }}d\theta sin\theta \overset{a}{\underset{0}{\int }}drr^{2}\Psi_{1,l+1,t}\left(r,\theta ,\varphi \right)\;\Psi_{1,l,t}\left(r,\theta ,\varphi \right)\;\right|}^{2}\delta \left(\hbar \omega -\hbar \omega_{1,l+1;1,l}\left(a\right)\right).\;$$

In (18), *C* was the quantity proportional to the square of the modulus of the matrix element of the dipole moment, taken for the Bloch functions of the conduction band of QD and *ω* was the frequency of the emitting light, the radiation energy $\;\hbar \omega_{1,l+1;1,l}$. (*a*) = $\;E_{1,l+1}\left(a\right)-E_{1,l}\left(a\right).$ After integration in (18), taking into account (7)–(10), we can obtain expressions that describe the intensities $\;I_{1,l,t}^{1,l+1,t}\left(a\right)$ of the dipole-allowed electron transitions between the quantum-confined states (n=1,l,t) and (n=1,l+1,t) (where l≤2, t=0, 1) in QDs:

(19) $${Ι}_{1,0,0}^{1,1,1}\left(\alpha \right)=\frac{3C\pi^{2}}{2^{5}{Χ}_{\alpha }^{2}j_{2}^{2}\left({Χ}_{\alpha }\right)}{\left(ci\left({Χ}_{\alpha }-\pi \right)-ci\left({Χ}_{\alpha }+\pi \right)+\frac{2\pi \left(1-\cos{Χ}_{\alpha }\right)}{\left({Χ}_{\alpha }^{2}-\pi^{2}\right)}\right)}^{2}$$

(20) $${Ι}_{1,1,1}^{1,2,1}\left(\alpha \right)=\frac{15C}{2^{9}{Χ}_{\alpha }^{2}{Χ}_{\beta }^{2}j_{2}^{2}\left({Χ}_{\alpha }\right)j_{3}^{2}\left({Χ}_{\beta }\right)}{\left({Κ}_{1}\left(a\right)+{Κ}_{2}\left(a\right)\right)}^{2}$$

(21) $${Κ}_{1}\left(a\right)=\frac{1}{2{Χ}_{\alpha }^{2}}[-2\sin X_{\alpha }\sin X_{\beta }-2{Χ}_{\beta }\sin X_{\alpha }\cos X_{\beta }-2\left({Χ}_{\alpha }-2\right)\sin X_{\beta }\cos X_{\alpha }+4{Χ}_{\beta }\left({Χ}_{\alpha }-1\right)+{Χ}_{\alpha \beta }\left({Χ}_{\alpha \beta }-2\right)ci{Χ}_{\alpha \beta }-\Delta {Χ}_{\alpha \beta }\left(\Delta {Χ}_{\alpha \beta }+2\right)ci\Delta {Χ}_{\alpha \beta }]$$

(22) $${Κ}_{2}\left(a\right)=\frac{\Delta {Χ}_{\alpha \beta }ci\Delta {Χ}_{\alpha \beta }+2\sin X_{\beta }\cos{Χ}_{\alpha }-2{Χ}_{\alpha }-{Χ}_{\alpha \beta }ci{Χ}_{\alpha \beta }}{{Χ}_{\alpha }{Χ}_{\beta }}+\frac{ci{Χ}_{\alpha \beta }-ci\Delta {Χ}_{\alpha \beta }}{2{Χ}_{\alpha }}+\frac{ci{Χ}_{\alpha \beta }-ci\Delta {Χ}_{\alpha \beta }}{{Χ}_{\beta }}+\frac{{Χ}_{\beta }-{Χ}_{\beta }\cos{Χ}_{\alpha }\cos\beta -{Χ}_{\alpha }\sin{Χ}_{\alpha }\sin{Χ}_{\beta }\;}{{Χ}_{\beta }^{2}-{Χ}_{\alpha }^{2}}$$

(23) $${Ι}_{1,2,0}^{1,3,1}\left(a\right)=\frac{2^{4}3^{2}C}{35{Χ}_{\beta }^{2}{Χ}_{\sigma }^{2}j_{3}^{2}\left({Χ}_{\beta }\right)j_{4}^{2}\left({Χ}_{\sigma }\right)}{\left({Κ}_{3}\left(a\right)+{Κ}_{4}\left(a\right)+{Κ}_{5}\left(a\right)+{Κ}_{6}\left(a\right)\right)}^{2}$$

(24) $${Κ}_{3}\left(a\right)=\Delta {Χ}_{\beta \sigma }\left(\Delta {Χ}_{\beta \sigma }^{2}+2\right)\sin\Delta {Χ}_{\beta \sigma }+\left(\Delta {Χ}_{\beta \sigma }^{2}-6\right)\cos\Delta {Χ}_{\beta \sigma }+\Delta {Χ}_{\beta \sigma }^{4}\left(ci\Delta {Χ}_{\beta \sigma }-1\right)-{Χ}_{\beta \sigma }\left({Χ}_{\beta \sigma }^{2}+2\right)\sin{Χ}_{\beta \sigma }-\left({Χ}_{\beta \sigma }^{2}-6\right)\cos\Delta {Χ}_{\beta \sigma }+{Χ}_{\beta \sigma }^{4}\left(ci{Χ}_{\beta \sigma }+1\right)+\frac{1}{{Χ}_{\beta }^{2}}+\frac{{Χ}_{\beta \sigma }\cos{Χ}_{\beta \sigma }+\left(2-{Χ}_{\beta \sigma }^{2}\right)\sin{Χ}_{\beta \sigma }+{Χ}_{\beta \sigma }^{3}\left(ci{Χ}_{\beta \sigma }+1\right)}{{Χ}_{\beta }^{2}{Χ}_{\sigma }^{2}}+\frac{{Χ}_{\beta \sigma }\cos{Χ}_{\beta \sigma }+\left(2-{Χ}_{\beta \sigma }^{2}\right)\sin{Χ}_{\beta \sigma }+{Χ}_{\beta \sigma }^{3}\left(ci{Χ}_{\beta \sigma }+1\right)}{{Χ}_{\sigma }^{2}}\;$$

(25) $${Κ}_{4}\left(a\right)=\left(\Delta {Χ}_{\beta \sigma }^{2}-2\right)\sin\Delta {Χ}_{\beta \sigma }-\Delta X_{\beta \sigma }\cos\Delta {Χ}_{\beta \sigma }-\Delta {Χ}_{\beta \sigma }^{3}\left(ci\Delta {Χ}_{\beta \sigma }+1\right)+\frac{3}{2}\frac{\cos\Delta {Χ}_{\beta \sigma }-\Delta {Χ}_{\beta \sigma }\sin\Delta {Χ}_{\beta \sigma }+\Delta {Χ}_{\beta \sigma }^{2}\left(ci\Delta {Χ}_{\beta \sigma }+1\right)-\cos{Χ}_{\beta \sigma }+{Χ}_{\beta \sigma }\sin{Χ}_{\beta \sigma }-{Χ}_{\beta \sigma }^{2}\left(ci{Χ}_{\beta \sigma }+1\right)}{{Χ}_{\beta }^{2}{Χ}_{\sigma }}$$

(26) $$K_{5}\left(a\right)=\frac{-\sin{Χ}_{\beta \sigma }+{Χ}_{\beta \sigma }\left(ci{Χ}_{\beta \sigma }+1\right)+\sin^{2}\left(\Delta {Χ}_{\beta \sigma }\right)-\Delta {Χ}_{\beta \sigma }\left(ci\Delta {Χ}_{\beta \sigma }+1\right)}{{Χ}_{\beta }^{2}}+\frac{\Delta {Χ}_{\beta \sigma }\cos\Delta {Χ}_{\beta \sigma }+\left(2-\Delta {Χ}_{\beta \sigma }^{2}\right)\sin\Delta {Χ}_{\beta \sigma }+\Delta {Χ}_{\beta \sigma }^{3}\left(ci\Delta {Χ}_{\beta \sigma }+1\right)+{Χ}_{\beta \sigma }\cos{Χ}_{\beta \sigma }+\left(2-{Χ}_{\beta \sigma }^{2}\right)\sin{Χ}_{\beta \sigma }}{{Χ}_{\beta }{Χ}_{\sigma }^{3}}$$

(27) $$K_{6}\left(a\right)={Χ}_{\beta \sigma }^{3}\left(ci\Delta {Χ}_{\beta \sigma }+1\right)+3\frac{-\sin\Delta {Χ}_{\beta \sigma }-\Delta {Χ}_{\beta \sigma }\cos\Delta {Χ}_{\beta \sigma }-\Delta {Χ}_{\beta \sigma }\sin\Delta {Χ}_{\beta \sigma }-\sin{Χ}_{\beta \sigma }-{Χ}_{\beta \sigma }\cos{Χ}_{\beta \sigma }-{Χ}_{\beta \sigma }^{2}\sin{Χ}_{\beta \sigma }}{{Χ}_{\beta }{Χ}_{\sigma }^{2}}+3\frac{\left(\Delta {Χ}_{\beta \sigma }+{Χ}_{\beta \sigma }\right)\left(ci\Delta {Χ}_{\beta \sigma }+1\right)-\sin\Delta {Χ}_{\beta \sigma }-\sin{Χ}_{\beta \sigma }}{{Χ}_{\beta }{Χ}_{\sigma }}-\frac{ci{Χ}_{\beta \sigma }+ci\Delta {Χ}_{\beta \sigma }}{{Χ}_{\beta \sigma }}+3\frac{-sin{Χ}_{\beta \sigma }+sin\Delta {Χ}_{\beta \sigma }+{Χ}_{\beta \sigma }ci{Χ}_{\beta \sigma }-\Delta {Χ}_{\beta \sigma }ci\Delta {Χ}_{\beta \sigma }+2{Χ}_{\beta }}{{Χ}_{\sigma }^{2}}+\frac{\cos{Χ}_{\beta \sigma }-\cos\Delta {Χ}_{\beta \sigma }+\Delta {Χ}_{\beta \sigma }\sin\Delta {Χ}_{\beta \sigma }-{Χ}_{\beta \sigma }\sin{Χ}_{\beta \sigma }+{Χ}_{\beta \sigma }^{2}ci{Χ}_{\beta \sigma }-\Delta {Χ}_{\beta \sigma }^{2}ci\Delta {Χ}_{\beta \sigma }}{2{Χ}_{\sigma }^{3}}+3\frac{ci{Χ}_{\beta \sigma }-ci\Delta {Χ}_{\beta \sigma }}{{Χ}_{\sigma }^{3}}+\frac{2-\cos{Χ}_{\beta \sigma }-\cos\Delta {Χ}_{\beta \sigma }}{2}+\frac{2{Χ}_{\sigma }-\Delta {Χ}_{\beta \sigma }\cos{Χ}_{\beta \sigma }-{Χ}_{\beta \sigma }\cos\Delta {Χ}_{\beta \sigma }}{2\left({Χ}_{\sigma }^{2}-{Χ}_{\beta }^{2}\right)}$$

where *ci*(*y*) is the integral cosinus.

### 2.4. Optical Absorption on Quantum-Confined Electron States in a Nanosystem

The cross section of light absorption $\sigma_{abs}\left(\omega ,a\right)$ on the spherical surface of a QD of radius a can be expressed in terms of its polarizability$\;A^{\prime \prime }\left(\omega ,a\right)$ [30]:

(28) $$\sigma_{abs}(\omega ,\;a)=4\pi (\omega /c)|A^{\prime \prime }\left(\omega ,a\right)|$$

where *ω* is the frequency of absorbed light and *c* is the speed of light in a vacuum. When condition (3) is satisfied, as well as (1) for slightly broadened electron states, for which the widths $\;\Gamma_{n,l}(a$) of the quantum-confined levels (*n*, *l*) (1) are small compared with frequencies $\;\omega_{n,l}\left(a\right),\;$i.e., at

(29) $$\Gamma_{n,l}(a)\;\ll \omega_{n,l}\left(a\right)$$

then QD can be represented as a single superatom [25] (where $\;\omega_{1,l}$(*a*) = $E_{1,l}\left(a\right)/\hbar$ is the frequency corresponding to the electron quantum-confined energy level $\;E_{1,l}\left(a\right)\;\left(1\right)).$ In this case, the polarizability $A^{\prime \prime }\left(\omega ,a\right)$ of QD was described by the formula [31]:

(30) $$\;A^{\prime \prime }\left(\omega ,\;a\right)=\frac{e^{2}}{m_{e\;}}\underset{n,l}{\sum }f_{n,l,t}^{n,l+1,t}\left[\frac{\omega_{n,l+1}^{2}\left(a\right)-\omega^{2}}{{\left(\omega_{n,l+1}^{2}\left(a\right)-\omega^{2}\right)}^{2}+{\left(\omega \Gamma_{n,l+1}\right)}^{2}}+i\frac{\omega \Gamma_{n,l+1}}{{\left(\omega_{n,l+1}^{2}\left(a\right)-\omega^{2}\right)}^{2}+{\left(\omega \Gamma_{n,l+1}\right)}^{2}}\right]$$

In (30), the oscillator strengths of the allowable transitions $f_{n,l,t}^{n,l+1,t}$(*a*) between the quantum-confined states of an electron (n=1,l,t) and (n=1,l+1,t) were defined by formula (17).

Let us consider the behavior of QDs in weak optical fields. Let us assume that in these fields, the polarizability $\;A^{\prime \prime }\left(\omega ,a\right)$ (30) of the QD was caused to only one electron transition between the quantum-confined states (n=1,l,t) and (n=1,l+1,t). In this case, the polarizability (30) of the QD is determined by the expression:

(31) $$\;\;A^{\prime \prime }\left(\omega ,a\right)=f_{n,l}^{n,l+1}\left(a\right)\frac{e^{2}}{m_{e\;}}\left[\frac{\omega_{n,l+1}^{2}\left(a\right)-\omega^{2}}{{\left(\omega_{n,l+1}^{2}\left(a\right)-\omega^{2}\right)}^{2}+{\left(\omega \Gamma_{n,l+1}\right)}^{2}}+i\frac{\omega \Gamma_{n,l+1}}{{\left(\omega_{n,l+1}^{2}\left(a\right)-\omega^{2}\right)}^{2}+{\left(\omega \Gamma_{n,l+1}\right)}^{2}}\right]$$

Consider the case in which the frequency of light *ω* is significantly lower and far from the resonant electron state (*n*, *l* + 1) frequency $\omega_{n,l+1}\left(a\right)\;(i.e.,\;\omega^{2\;}\ll {\left(\omega_{n,l+1}\left(a\right)\right)}^{2\;}.$ Taking into account (1) and (17), we write the polarizability (31) as

(32) $$A_{1}^{\prime \prime }\left(a\right)\approx 4\left(m_{e\;}/m_{o\;}\right)(X_{1,l+1\;}{}^{2}-X_{1,l\;}{}^{2})\;{\left(X_{n,l+1}\right)}^{-4}\;{\left|D_{1,l,t}^{1,l+1,t}\left(a\right)/ea\right|}^{2}\;{\left(a/a_{o\;}\right)}^{4}\;{\left(a_{o}\right)}^{3}$$

where $a_{o\;}=$ 0.053 nm is the Bohr radius of a free electron. We neglect the real part of the polarizability (31) QD for frequencies close to resonant $\omega \approx \omega_{n,l+1}\left(a\right)$, since it is much smaller than the imaginary part. As a result, we obtain an expression that describes the polarizability of QD:

(33) $$A_{2}^{\prime \prime }\left(a\right)\approx i\;4\;(X_{1,l+1\;}{}^{2}-X_{1,l\;}{}^{2})\;{\left(X_{n,l+1}\right)}^{-2}\;(Ry_{o\;}/\hbar \Gamma_{n,l+1}){\left|D_{1,l,t}^{1,l+1,t}\left(a\right)/ea\right|}^{2}{\left(a/a_{o\;}\right)}^{2}\;{\left(a_{o}\right)}^{3}$$

where $Ry_{o\;}=$13.606 eV is the Rydberg constant. In the case $\omega^{\;}\gg \omega_{1,l+1}$ $(i.e.,\;\omega^{2\;}\gg {\left(\omega_{n,l+1}\left(a\right)\right)}^{2\;}$, the polarizability of QD is given by a negative real part:

(34) $$A_{3}^{\prime \prime }\left(a\right)\approx -4{\left(Ry_{o\;}/E_{\omega }\right)}^{2}\;(X_{1,l+1\;}{}^{2}-X_{1,l\;}{}^{2}){\left|D_{1,l,t}^{1,l+1,t}\left(a\right)/ea\right|}^{2}\left(m_{0\;}/m_{e\;}\right)\;{\left(a_{o}\right)}^{3}$$

where $E_{\omega }=\hbar \omega .$

Using expressions (32)–(34), we write the ratios of the polarizabilities QD absolute values as:

(35) $$A_{1}^{\prime \prime }\left(a\right)/A_{2}^{\prime \prime }\left(a\right)=\hbar \Gamma_{n,l+1}/E_{n,l+1}\ll 1$$

(36) $$A_{3}^{\prime \prime }\left(a\right)/A_{1}^{\prime \prime }\left(a\right)={\left(E_{n,l+1}/E_{\omega }\right)}^{2}\ll 1$$

## 3. Numerical Results and Discussion

The behavior we considered of the quantum-confined states of an electron arising in QD in the field of a light wave are applicable to an ensemble of non-interacting QDs, i.e., to the QD ensemble with the QD concentration *N* [30,31]:

(37) $$a\;N^{1/3}\ll 1$$

Let us carry out numerical estimates of dipole moments of allowable intraband transitions $D_{1,l,t}^{1,l+1,t}$ (4), oscillator strengths of allowable intraband transitions $f_{1,l,t}^{1,l+1,t}$ (17), and intensities $\;I_{1,l,t}^{1,l+1,t}\;$(18) of dipole-allowed intraband electron transitions between the quantum-confined states (n=1,l,t) and (n=1,l+1,t) (where l≤2, t=0, 1) in spherical QDs with radius *a* = 5.5 nm, containing the perovskites ${\mathrm{FAPbBr}}_{3\;}$ (for l=0,1) (see Table 1) and $\left\{\mathrm{en}\right\}{\mathrm{FAPbBr}}_{3\;}$ (for *l* =0,1,2) (see Table 2). From Formulas (4)–(6) and (11)–(27) it follows that the values of the dipole moments of allowable transitions $D_{1,l,t}^{1,l+1,t}$ (4), oscillator strengths of allowable transitions $f_{1,l,t}^{1,l+1,t}$ (17), as well as the intensities $\;I_{1,l,t}^{1,l+1,t}\;$(18) of dipole-allowed electron transitions with increasing azimuthal quantum number *l* (from 0 to 1) for the perovskites ${\mathrm{FAPbBr}}_{3\;},\;$and *l* (from 0 to 2) for the perovskites $\left\{\mathrm{en}\right\}{\mathrm{FAPbBr}}_{3\;})$ decrease in proportion to the coefficient ${\left(X_{1,l+1\;}{}^{2}-X_{1,l\;}{}^{2}\right)}^{-1}$ (where $\;\;X_{1,0}=\pi ;\;X_{1,1}$ = 4.493; $X_{1,2}$ = 5.763; $X_{1,3}=$6.988 are the roots of the Bessel function, i.e.,$\;J_{l+1/2}$ ($X_{n,l})$ = 0 [33]). In this case, the values of the dipole moments $D_{1,l,t}^{1,l+1,t}$ (4) in the QD perovskites $\left\{\mathrm{en}\right\}{\mathrm{FAPbBr}}_{3\;}$(for *l* ≤2) will exceed the corresponding values of dipole moments in QD perovskites ${\mathrm{FAPbBr}}_{3\;}\left(\mathrm{for}\;l=0,1\right)\;$(see Table 1 and Table 2). This is due to the fact that according to Formulas (5) and (6) of dipole moments, (4) are proportional to the coefficient *Λ* (6). The coefficient *Λ* (6) is inversely proportional to the permittivity *ε* QDs. Since in NC perovskites ${\mathrm{FAPbBr}}_{3\;}$ the permittivity *ε* = 8.6 is greater than the value *ε* = 7 in QD perovskites $\left\{\mathrm{en}\right\}{\mathrm{FAPbBr}}_{3\;}$, the coefficient Λ = $3^{-1}$ for the QD perovskites $\left\{\mathrm{en}\right\}{\mathrm{FAPbBr}}_{3\;}$ will exceed Λ = 0.28 in the QD perovskites ${\mathrm{FAPbBr}}_{3\;}$.

The values of the dipole moments $D_{1,l,t}^{1,l+1,t}$, according to Formulas (11)–(16), as well as of oscillator strengths of allowable transitions $f_{1,l,t}^{1,l+1,t}$ (17) with increasing azimuthal quantum number *l* (from 0 to 1), decrease from $D_{1,0,0}^{1,1,1}=$ 8.4$\;D_{0}$ to $D_{1,1,1}^{1,2,0}=$ 5.7 $\;D_{0}$ (where $D_{0}=eÅ$ in Debye units) and from $f_{1,0,0}^{1,1,1}=$ 0.24 to $f_{1,1,1}^{1,2,0}=$ 0.14 in the QD perovskites ${\mathrm{FAPbBr}}_{3\;}$(see Table 1), and also decrease from $D_{1,0,0}^{1,1,1}=$ 9.9 to $D_{1,2,0}^{1,3,1}=$ 2.96 and from $f_{1,0,0}^{1,1,1}=$ 0.33 to $f_{1,2,0}^{1,3,1}=$ 0.045 in the QD perovskites $\left\{\mathrm{en}\right\}{\mathrm{FAPbBr}}_{3\;}$with increasing *l* (from 0 to 2) (see Table 2). As the azimuthal quantum number *l* increases (from 0 to 2), the intensities $\;I_{1,l,t}^{1,l+1,t}\;$(17) of the dipole-allowed electron transitions in the QD perovskites $\left\{\mathrm{en}\right\}{\mathrm{FAPbBr}}_{3\;}$ also decrease from $I_{1,0,0}^{1,1,1}=$0.65 to $I_{1,2,0}^{1,3,1}=0.20\;$(see Table 2). The intensity values (16) in in the QD perovskites ${\mathrm{FAPbBr}}_{3\;}$decrease from $I_{1,0,0}^{1,1,1}=$0.65 to $I_{1,1,1}^{1,2,0}=0.31$ as *l* decreases from 0 to 1 (see Table 1).

Table 3 and Table 4 show the numerical values of polarizabilities $\;A_{1}^{\prime \prime }\left(a\right)$ (32) (in case, when the frequency of light *ω* is significantly lower and far from the resonant electron state (n, *l* + 1) frequency $\omega_{n,l+1}\left(a\right),\;$at ${\left(\omega /\omega_{1,l+1}\right)}^{2\;}=10^{-2}$), as well as the absorption cross sections $\;\sigma_{abs}$(*ω*, *a*) (28), which were due to dipole-allowed electron transitions between the quantum-confined states (n=1,l,t) and (n=1,l+1,t) (where l≤2, t=0, 1) in spherical QDs with radius *a* = 5.5 nm containing the perovskites ${\mathrm{FAPbBr}}_{3\;}$ (for *l* = 0, 1) and $\left\{\mathrm{en}\right\}{\mathrm{FAPbBr}}_{3\;}\;$(for *l* ≤ 2). Polarizabilities $\;A_{1}^{\prime \prime }$ (32), as well as the corresponding absorption cross sections $\;\sigma_{abs}$(*ω*,*a*) (28), according to Formulas (11)–(17), with increasing azimuthal quantum number *l* (from 0 to 1) for the perovskites ${\mathrm{FAPbBr}}_{3\;}$, and *l* (from 0 to 2) for the perovskites {en}${\mathrm{FAPbBr}}_{3\;}$decrease in proportion to the coefficient ${\left(X_{1,l+1\;}{}^{2}-X_{1,l\;}{}^{2}\right)}^{-1}{\left(X_{n,l+1}\right)}^{-4}.$ The values of polarizabilities $\;A_{1}^{\prime \prime }$ (32) and absorption cross sections $\;\sigma_{abs}$(*ω*, *a*) (28) with increasing *l* (from 0 to 1) decrease from$\;\;A_{1}^{\prime \prime }=1.1\cdot 10^{-20}{\mathrm{cm}}^{3}\;$(and $\sigma_{abs}=6.5\cdot 10^{-17}{\mathrm{cm}}^{2})$ to$\;\;A_{1}^{\prime \prime }=5.4\cdot 10^{-21}{\mathrm{cm}}^{3}\;$(and$\;\sigma_{abs}=5.5\cdot 10^{-17}{\mathrm{cm}}^{2}\;$in the QD perovskites ${\mathrm{FAPbBr}}_{3\;}$(see Table 3), and also decrease from $\;A_{1}^{\prime \prime }=8.56\cdot 10^{-21}{\mathrm{cm}}^{3}\;$(and $\sigma_{abs}=6.7\cdot 10^{-17}{\mathrm{cm}}^{2})$ to $\;A_{1}^{\prime \prime }=2.8\cdot 10^{-22}{\mathrm{cm}}^{3}$ (and $\sigma_{abs}=4.35\cdot 10^{-18}{\mathrm{cm}}^{2})$ in the QD perovskites $\left\{\mathrm{en}\right\}{\mathrm{FAPbBr}}_{3\;}$ with increasing *l* (from 0 to 2) (see Table 4). Since the polarizabilities (31) and absorption cross sections (28) are proportional to the ratio $\left(m_{e\;}/m_{o\;}\right)$, and the effective mass of the electron in the QD perovskites ${\mathrm{FAPbBr}}_{3\;}$ is greater than the effective mass of the electron in the QD perovskites {en}${\mathrm{FAPbBr}}_{3\;}$, the values of polarizabilities $\;A_{1}^{\prime \prime }$ (32) and absorption cross sections $\;\sigma_{abs}$(*ω*, *a*) (28) in in the QD perovskites ${\mathrm{FAPbBr}}_{3\;}$ exceed the corresponding values of polarizabilities (32) and absorption cross sections (28) in the QD perovskites {en}${\mathrm{FAPbBr}}_{3\;}$(see Table 3 and Table 4). The frequencies $\omega_{1,l}\left(a\right)$ are in the infrared region.

Table 3 and Table 4 show the numerical values of polarizabilities$\;A_{2}^{\prime \prime }\left(a\right)$ (33) and absorption cross sections $\;\sigma_{abs}$(*ω*, *a*) (28) in the QD perovskites ${\mathrm{FAPbBr}}_{3\;}$, as well as the values of polarizabilities (33) and absorption cross sections (28) in the QD perovskites {en}${\mathrm{FAPbBr}}_{3\;}$ at resonant absorption of light with frequencies $\omega \approx \omega_{1,l+1}\left(a\right)$. These values, according to formulas (12)–(17) (35) (for the ratio ($(\hbar \Gamma_{1,l+1}/E_{1,l+1}$) =$\;10^{-2}$), are three orders of magnitude higher than the corresponding values of polarizabilities (32) and absorption cross sections (28) in case (${\left(\omega /\omega_{1,l+1}\right)}^{2\;}=10^{-2}$).

According to Formula (36), the values of polarizabilities$\;A_{3}^{\prime \prime }\left(a\right)$ (34) and absorption cross sections $\;\sigma_{abs}$(*ω*, *a*) (28) in the QD perovskites ${\mathrm{FAPbBr}}_{3\;}$and QD perovskites {en}${\mathrm{FAPbBr}}_{3\;}$for frequencies $\omega^{\;}$(for ${\left(\omega /\omega_{1,l+1}\right)}^{2\;}=10^{2}$) will be two orders of magnitude smaller than the corresponding values of polarizabilities (32) and absorption cross sections (28) in the case(${\left(\omega /\omega_{1,l+1}\right)}^{2\;}=10^{-2}$) (see Table 3 and Table 4).

It should be noted that the energy levels (1) of an electron in the QD perovskites ${\mathrm{FAPbBr}}_{3\;}$ and in the QD perovskites $\left\{\mathrm{en}\right\}{\mathrm{FAPbBr}}_{3\;}$ satisfied conditions (2) and (3). Therefore, the values of oscillator strengths transitions (17), polarizabilities (30), and light absorption (28) were obtained under the assumption that the conduction bands in perovskite QDs had a parabolic shape.

The optical attenuation coefficient ***γ***(*ω*, *a*) of light, due to both the absorption and scattering of light by quantum-confined states electron (*n*, *l*) (1) in the QDs perovskite ${\mathrm{FAPbBr}}_{3\;}$ and QDs perovskite {en}${\mathrm{FAPbBr}}_{3\;}$ of radius *a* is determined by the expression [30]:

(38) $$\gamma (\omega ,\;a)=\;N\;(\sigma_{abs}(\omega ,a)+\sigma_{sc}(\omega ,a))$$

where *N* is the concentrations of the QDs perovskite ${\mathrm{FAPbBr}}_{3\;}$ and QDs perovskite $\{en\}{\mathrm{FAPbBr}}_{3\;}$in the nanosystem. Formula (38) was obtained for an ensemble of non-interacting QDs. In this case, condition (37) is satisfied.

Formula (38) includes the cross section for the scattering$\;\sigma_{sc}$(*ω*, *a*) of light by a QD. We can write the expression for the cross section $\;\sigma_{sc}$(*ω*, *a*) of elastic scattering of the electromagnetic wave with frequency *ω* by the QD of radius *a* [30] as:

(39) $$\sigma_{sc}(\omega ,\;a)=2^{7}\;3^{-3}\;\pi^{3}\;{\left(\omega /c\right)}^{4}\;{\left|A^{\prime \prime }\left(\omega ,a\right)\right|}^{2}$$

Formula (39) includes the cross section for the scattering$\;\sigma_{sc}\;$(*ω*,*a*) of light by a QD. Since, according to (39)$\;\;\sigma_{sc}\;$(*ω*,*a*)$\sim \;\left|A^{\prime \prime }\left(\omega ,a\right)\right|{\;}^{2},$ the values of the scattering cross section $\;\sigma_{sc}\;$(*ω*,*a*) (39) are six orders of magnitude smaller than the corresponding values $\;\sigma_{abs}$(*ω*,*a*) (28). Therefore, to estimate the optical attenuation coefficient *γ*(*ω*, *a*) (38), the processes of light scattering are not taken into account.

Thus, nanosystems containing the QDs perovskite ${\mathrm{FAPbBr}}_{3\;}$and the QDs perovskite $\{en\}{\mathrm{FAPbBr}}_{3\;}$are highly absorbing media in the infrared range. In nanosystems containing the QDs perovskite${\;\mathrm{FAPbBr}}_{3\;}$and the QDs perovskite $\{en\}{\mathrm{FAPbBr}}_{3\;}$with a concentration of QDs *N*$\;\approx (10^{14}-10^{16}$) ${\mathrm{cm}}^{-3}$ [12,13], the optical attenuation coefficient (38) coefficient takes on a significant value *γ*(*ω, a*) ≈ (1$-10^{2}$) ${\mathrm{cm}}^{-1}.$The increase of the absorption due QDs application is of high significance in perovskite solar cells. As was proven in [26], the strengthening of the sun-light absorption by the use of nano-components leads to an increase of the overall efficiency of solar cells. In p-n junction cells, quantum coupling of nano-localized plasmons to band electrons can even double the photo-efficiency just by the increase of the absorption [21]. In perovskite cells this channel is, however, ineffective [27,28], and localized plasmons contribute rather to internal electrical phenomena in cells. Application of QDs simultaneously with metallic nanoparticles would activate both channels simultaneously—absorption and electrical.

## 4. Conclusions

Optical properties of nanosystems containing the QDs perovskite ${\;\mathrm{FAPbBr}}_{3\;}$and QDs $\{en\}{\mathrm{FAPbBr}}_{3\;}$in weak optical fields result in only single intraband electron transitions between the quantum-confined states. In this case, nanosystems are highly absorbing media in the infrared region in weak optical fields. The obtained results can be used for creating the nano- and heterostructures for advanced nanophotonic applications that operate in conditions of weak optical fields in the infrared region.

## Figures and Tables

**Table 1:** The estimated values of oscillator strength $f_{1,l,t}^{1,l+1,t}$ (17), transition dipole moments $\;D_{1,l,t}^{1,l+1,t}$ (4) (where $D_{0}=eÅ$ in Debye units), and radiation intensity$\;I_{1,l,t}^{1,l+1,t}$ (18) caused by dipole-allowed electron transitions between the quantum-confined states (n=1, l,t)→(n=1, l+1,t) (where *l* = 0, 1 and t = 0, 1) in the QD perovskites ${\mathrm{FAPbBr}}_{3\;}$ with radius a=5.5 nm.

(1, l, t) → (1, l+1, t)	$f_{1,l,t}^{1,l+1,t}$	$\;\;D_{1,l,t}^{1,l+1,t}$ $\;(D_{0})$	$\;\;I_{1,l,t}^{1,l+1,t}$
(1,0,0) → (1,1,1)	0.24	8.4	0.65
(1,l,1) → (1,2,0)	0.14	5.7	0.31

**Table 2:** The estimated values of oscillator strength $f_{1,l,t}^{1,l+1,t}$ (17) transition dipole moments$\;D_{1,l,t}^{1,l+1,t}$ (3) (where $D_{0}=eÅ$ in Debye units) and radiation intensity$\;I_{1,l,t}^{1,l+1,t}$ (18) caused by dipole-allowed electron transitions between the quantum-confined states (n=1, l,t)→(n=1, l+1,t) (where *l* = 0, 1, 2 and t = 0, 1) in the QD perovskites $\left\{\mathrm{en}\right\}{\mathrm{FAPbBr}}_{3\;}$ with radius a=5.5 nm.

	$f_{1,l,t}^{1,l+1,t}$	$\;\;D_{1,l,t}^{1,l+1,t}$ $\;(D_{0})$	$\;\;I_{1,l,t}^{1,l+1,t}$
(1,0,0) → (1,1,1)	0.33	9.9	0.65
(1,l,1) → (1,2,0)	0.194	6.72	0.31
(1,2,0) → (1,3,1)	0.045	2.96	0.20

**Table 3:** The estimated values of polarizabilities $\;A_{1}^{''}\left(a\right)$ (32) ($\mathrm{for}\;{\left(\omega /\omega_{1,l+1}\right)}^{2\;}=10^{-2}$ )), $\;A_{2}^{''}\left(a\right)$ (33) (for $\approx \omega_{1,l+1}\left(a\right)$ ), $\;A_{3}^{''}\left(a\right)$ (34) (for ${\left(\omega /\omega_{1,l+1}\right)}^{2\;}=10^{-2}$ ) (where *ω* is the frequency of the absorbed light and the resonant electron state (n, *l*+ 1) frequency $\omega_{n,l+1}\left(a\right)$ ), as well as the corresponding absorption cross sections $\;\sigma_{abs}$ (*ω*, *a*) (28) caused by dipole-allowed electron transitions between the quantum-confined states (n=1, l,t)→(n=1, l+1,t) (where *l*= 0, 1 and t = 0, 1) in the QD perovskites ${\mathrm{FAPbBr}}_{3\;}$ with radius a=5.5 nm.

(1, l, t) → (1, l+1, t)	${\left(\omega /\omega_{1,l+1}\right)}^{2\;}$	$\left|A^{\prime \prime }\left(\omega ,a\right)\right|$ $\;(10^{-22}cm^{3})$	$\sigma_{abs}$ $\;(10^{-22}cm^{2})$
(1,0,0) → (1,1,1)	$10^{-2}$	$1.1\;\cdot 10^{2}$	$6.5\;\cdot 10^{5}$
(1,0,0) → (1,1,1)	1	$1.1\cdot 10^{4}$	$7.1\cdot 10^{8}$
(1,0,0) → (1,1,1)	$10^{2}$	1	$6.3\;\cdot 10^{5}$
(1,1,1) → (1,2,0)	$10^{-2}$	$5.4\;\cdot 10^{1}$	$5.5\;\cdot 10^{5}$
(1,1,1) → (1,2,0)	1	$3.9\;\cdot 10^{3}$	$4\cdot 10^{8}$
(1,1,1) → (1,2,0)	$10^{2}$	$6\;\cdot 10^{-1}$	$6\cdot 10^{5}$

**Table 4:** The estimated values of of polarizabilities $\;A_{1}^{''}\left(a\right)$ (32) ($\mathrm{for}\;{\left(\omega /\omega_{1,l+1}\right)}^{2\;}=10^{-2}$ )), $\;A_{2}^{''}\left(a\right)$ (33) (for $\approx \omega_{1,l+1}\left(a\right)$ ), and $\;A_{3}^{''}\left(a\right)$ (33) (for ${\left(\omega /\omega_{1,l+1}\right)}^{2\;}=10^{-2}$ ) (where *ω* is the frequency of the absorbed light and the resonant electron state (n, *l*+ 1) frequency $\omega_{n,l+1}\left(a\right)$ ), as well as the corresponding absorption cross sections $\;\sigma_{abs}$ (*ω*, *a*) (28) caused by dipole-allowed electron transitions between the quantum-confined states (n=1, l,t)→(n=1, l+1,t) (where *l*= 0, 1, 2 and t = 0, 1) in the QD perovskites $\left\{\mathrm{en}\right\}{\mathrm{FAPbBr}}_{3\;}$ with radius a=5.5 nm.

(1, l, t) → (1, l+1, t)	${\left(\omega /\omega_{1,l+1}\right)}^{2\;}$	$\left|A^{\prime \prime }\left(\omega ,a\right)\right|$ $\;(10^{-22}cm^{3})$	$\sigma_{abs}$ $\;(10^{-22}cm^{2})$
(1,0,0) → (1,1,1)	$10^{-2}$	$8.56\cdot 10^{1}$	$6.7\cdot 10^{5}$
(1,0,0) → (1,1,1)	1	$9\cdot 10^{3}$	$7\cdot 10^{8}$
(1,0,0) → (1,1,1)	$10^{2}$	$8.4\;\cdot 10^{-1}$	$6.5\cdot 10^{5}$
(1,1,1) → (1,2,0)	$10^{-2}$	$4.4\;\cdot 10^{1}$	$5.5\cdot 10^{5}$
(1,1,1) → (1,2,0)	1	$3.2\cdot 10^{3}$	$4\cdot 10^{8}$
(1,1,1) → (1,2,0)	$10^{2}$	$2.6\;\cdot 10^{-1}$	$3.2\;\cdot 10^{5}$
(1,2,0) → (1,3,1)	$10^{-2}$	2.8	$4.35\cdot 10^{4}$
(1,2,0) → (1,3,1)	1	$2.6\;\cdot 10^{2}$	$1.5\cdot 10^{8}$
(1,2,0) → (1,3,1)	$10^{2}$	$2.8\;\cdot 10^{-2}$	$5.1\;\cdot 10^{4}$

## Data Availability

Not applicable.
